# Post-treatment serologic, cerebrospinal fluid, MRI, and salivary antibody responses in HIV-negative neurosyphilis: a retrospective study

**DOI:** 10.3389/fneur.2026.1939496

**Published:** 2026-09-29

**Authors:** Xuan Long, Weipeng Lin, Jiahui Shen, Xueying Zhong, Sichan Wu, Donghua Liu

**Affiliations:** Department of Dermatology and Venereology, First Affiliated Hospital of Guangxi Medical University, Nanning, China

**Keywords:** cerebrospinal fluid, MRI, neurosyphilis, salivary, *Treponema pallidum*

## Abstract

**Objectives:**

This study aimed to characterize longitudinal serologic, cerebrospinal fluid (CSF), MRI, and salivary antibody responses after treatment in HIV-negative neurosyphilis.

**Methods:**

We retrospectively analyzed 73 HIV-negative adults treated for neurosyphilis from June 2021 to June 2025. Survival endpoints were defined as time to first documented serologic response or CSF normalization. Paired baseline and 12-month saliva samples were available for 25 patients. Cox models were exploratory, and longitudinal MRI findings were analyzed descriptively.

**Results:**

Among 25 patients with paired saliva samples, salivary antibody OD decreased at 12 months (estimated paired decrease, 0.36 OD units; 95% CI, 0.14–0.64) and showed a modest correlation with the decline in serum TRUST titer (Spearman *ρ* = 0.396). Of 73 patients, 36 achieved a first documented serologic response. Higher baseline serum TRUST titer was associated with earlier documented response (adjusted HR per twofold increase, 1.34; 95% CI, 1.11–1.61), whereas elevated CSF protein was associated with later documented response (adjusted HR, 0.41; 95% CI, 0.20–0.86). Median times to first documented normalization were 12 months for CSF TRUST, 13 months for CSF WBC, and 20 months for CSF protein; these estimates were derived from separate endpoint-specific risk sets. Follow-up MRI findings in 13 clinically selected patients were descriptive.

**Conclusion:**

The post-treatment decline in salivary anti-*T. pallidum* antibody OD provides a preliminary non-invasive signal of treatment-associated change. Prospective validation in larger cohorts is required.

## Introduction

Neurosyphilis is a major central nervous system complication of *Treponema pallidum* infection and can occur at any stage of syphilis (1). Although it has historically been associated with late-stage manifestations such as general paresis and tabes dorsalis, the clinical spectrum of neurosyphilis has shifted in recent years. An increasing proportion of cases now present as meningeal or meningovascular disease or as asymptomatic CSF abnormalities, often affecting immunocompetent young and middle-aged adults (2).

Intravenous high-dose aqueous crystalline penicillin G is the recommended first-line therapy for neurosyphilis (3, 4). Despite well-established antimicrobial efficacy, evaluating treatment response remains challenging because serum non-treponemal titers and CSF WBC count, protein concentration, and non-treponemal tests may change at different times (5). In routine practice, follow-up assessments are also frequently performed at irregular intervals, complicating the interpretation of the observed time to response (6).

Neuroimaging abnormalities are common in neurosyphilis, including white matter lesions, cerebral atrophy, and vascular changes (7–9), yet their prognostic significance and their relationships with post-treatment serologic and CSF responses remain unclear (10).

Our team recently reported that salivary anti-*Treponema pallidum* antibodies detected by ELISA showed good diagnostic performance for identifying neurosyphilis (11). However, evidence of longitudinal post-treatment salivary antibody changes remains limited. We therefore investigated paired changes in salivary antibody OD signals as the principal novel exploratory objective. Serum TRUST and CSF follow-up findings were evaluated for clinical context, whereas longitudinal MRI observations were analyzed descriptively.

## Methods

### Study design and participants

This retrospective cohort included adults diagnosed with neurosyphilis at the First Affiliated Hospital of Guangxi Medical University between June 2021 and June 2025. Clinical, serum TRUST, CSF, salivary anti-*Treponema pallidum* antibody OD, and brain MRI data were extracted from hospital records. Eligibility required at least one post-treatment serum TRUST assessment within 3–24 months; available CSF assessments during the same period were included in endpoint-specific analyses. Endpoint-specific follow-up assessments are summarized in Supplementary Table S1. The institutional ethics committee approved the study (no. 2025-S0809).

Because no universally accepted diagnostic gold standard exists, neurosyphilis was diagnosed by treating specialists through an integrated assessment of neurological or psychiatric manifestations, serum syphilis serology, CSF TRUST, CSF TPPA, CSF WBC count, and CSF protein concentration (3, 12). A reactive CSF TRUST result or a CSF TPPA titer ≥1:320 was considered laboratory evidence supporting neurosyphilis (13). In patients who did not meet either criteria, compatible neurological manifestations together with CSF pleocytosis and/or elevated protein, after exclusion of a more likely alternative diagnosis, supported the diagnosis. Patients without compatible neurological or psychiatric manifestations were classified as asymptomatic.

Patients were excluded for incomplete baseline data, positive HIV testing, absence of post-treatment serum TRUST follow-up, recent antisyphilitic antibiotic exposure, or a more likely alternative neurological, infectious, inflammatory, vascular, or neoplastic diagnosis. Alternative causes were assessed through medical history, neurological examination, routine CSF findings, neuroimaging, and clinically indicated investigations; no uniform prospective exclusion panel was available.

### Treatment and follow-up

All patients received antisyphilitic treatment. Penicillin-based treatment consisted of intravenous aqueous crystalline penicillin G at 18–24 million units/day (3–4 million units every 4 h) for 14 days (3, 12). The non-penicillin regimen consisted of intravenous ceftriaxone 2 g once daily for 14 days (14).

Treatment completion was defined as time zero. For serum outcomes, event time was the date of the first post-treatment assessment documenting a fourfold or greater decline in serum TRUST titer from baseline or seroreversion (15, 16). Patients without a documented response were censored at their last available serum TRUST assessment. Because the endpoint was ascertainable only at observed visits, the estimand was the time to the first documented serologic response rather than the precise biological response time.

For CSF outcomes, event time was the date of the first post-treatment lumbar puncture documenting normalization of the corresponding parameter; patients without normalization were censored at their last available post-treatment lumbar puncture. Repeat lumbar punctures were determined by routine clinical care rather than a predefined schedule. Kaplan–Meier estimates were retained as descriptive estimates of the time to first documented normalization, not precise biological normalization time.

Baseline brain MRI was defined as an MRI performed before or at neurosyphilis diagnosis. Original MRI images and contemporaneous radiology reports were jointly reviewed by two study investigators (XL and WL), who categorized abnormalities according to lesion pattern and anatomical location. This was a consensus review rather than a blinded, independent dual-reader assessment. Repeat MRI was clinically indicated rather than systematically scheduled; longitudinal findings were therefore analyzed descriptively.

### Saliva sample collection and antibody measurement

Unstimulated whole saliva samples were collected before treatment and at the 12-month post-treatment follow-up when available. Participants were instructed not to eat, drink, smoke, chew gum, or perform oral hygiene for at least 1 h before sample collection. In the morning, 3–5 mL of unstimulated whole saliva was obtained by passive drooling into sterile tubes. Samples were centrifuged at 1800 × g for 15 min to remove cellular debris, and the supernatants were stored at −80 °C until analysis.

Salivary anti-*T. pallidum* antibodies were measured using a commercial enzyme-linked immunosorbent assay kit (*Treponema pallidum* Antibody Diagnostic Kit; Beijing Wantai Biological Pharmacy Enterprise Co., Ltd., Beijing, China), according to the manufacturer’s instructions. The assay used recombinant TP15, TP17, and TP47 antigens and detected anti-*T. pallidum* IgM/IgG signals. OD was read at 450 nm using a microplate reader. Salivary OD decline was defined as baseline OD minus follow-up OD.

### Outcome definitions

The primary survival outcome was time to first documented serologic response, defined as a fourfold or greater decline in serum TRUST titer from baseline or seroreversion (15, 16). Secondary survival outcomes were time to first documented CSF WBC normalization (≤5 cells/μL), CSF protein normalization (≤450 mg/L), CSF TRUST normalization (seroreversion or a fourfold or greater decline), and complete CSF normalization. Complete normalization required all three parameters to meet their normal criteria. Each endpoint was evaluated in its corresponding baseline-abnormal risk set with available post-treatment CSF follow-up.

### Statistical analysis

Continuous variables are presented as medians with interquartile ranges and categorical variables as counts and percentages. Between-group comparisons used the Mann–Whitney *U*-test, χ^2^ test, or Fisher’s exact test, as appropriate. Kaplan–Meier and log-rank methods were used to evaluate time to first documented serologic response and CSF normalization.

Cox regression evaluated factors associated with the first documented serologic response. Baseline serum TRUST titer was modeled per twofold increase. The exploratory multivariable model included baseline serum TRUST titer, CSF protein >450 mg/L, symptomatic status, and treatment regimen. The complete-case model included all 73 patients and 36 events; no imputation was performed. The proportional hazards assumption was assessed using Schoenfeld residuals. The availability and missingness of candidate covariates for Cox regression are reported in Supplementary Table S3.

Salivary analyses used the Wilcoxon signed-rank test for paired changes, the Mann–Whitney *U*-test for TRUST-stratum comparisons, and Spearman correlation for changes in salivary OD and serum TRUST titer. Hodges–Lehmann estimates with bootstrap 95% confidence intervals were reported. A baseline-adjusted linear model with HC3 robust standard errors was performed as a sensitivity analysis. All tests were two-sided, with *p* < 0.05 considered statistically significant.

## Results

### Characteristics of the study population

A total of 73 HIV-negative patients with neurosyphilis were included in this study (Table 1). During follow-up, 36 patients (49.3%) achieved a documented serologic response, whereas 37 patients (50.7%) were censored without a documented response. The median age of the overall cohort was 38.0 years (IQR, 29.0–54.0), and 51 patients (69.9%) were male. Penicillin-based therapy was received by 52 patients (71.2%).

**Table 1:** Baseline characteristics of patients with neurosyphilis according to serologic response.

Variables	Total (*n* = 73)	Non-response (*n* = 37)	Response (*n* = 36)	*p*-value
Age, median (Q1, Q3), years	38.00 (29.00, 54.00)	44.00 (33.00, 56.00)	34.50 (27.00, 50.25)	0.074
Sex, *n* (%)				0.800
Male	51 (69.9)	25 (67.6)	26 (72.2)	
Female	22 (30.1)	12 (32.4)	10 (27.8)	
Serum TRUST titer, median (Q1, Q3)	1:16 (1:8, 1:32)	1:8 (1:4, 1:32)	1:32 (1:16, 1:64)	<0.001
CSF TRUST titer, median (Q1, Q3)	0 (0, 1:1)	0 (0, 1:1)	0 (0, 1:1)	0.020
CSF WBC count, median (Q1, Q3), cells/μL	5.00 (0.00, 28.50)	3.00 (0.00, 20.00)	10.00 (2.00, 34.00)	0.052
CSF protein concentration, median (Q1, Q3), mg/L	515.30 (394.95, 656.10)	531.00 (412.90, 645.70)	495.10 (364.75, 666.30)	0.960
Salivary antibody OD, median (Q1, Q3)	0.86 (0.29, 1.61)	0.56 (0.26, 1.63)	0.89 (0.45, 1.55)	0.660
Late syphilis stage, *n* (%)	39 (53.4)	23 (62.2)	16 (44.4)	0.162
Baseline MRI abnormality, *n*/*N* (%)	36/63 (57.1)	17/35 (48.6)	19/28 (67.9)	0.200
Neurosyphilis subtype, *n* (%)				0.878
Tabes dorsalis	3 (4.1)	1 (2.7)	2 (5.6)	
General paresis	15 (20.5)	9 (24.3)	6 (16.7)	
Meningovascular neurosyphilis	8 (11.0)	4 (10.8)	4 (11.1)	
Meningitic neurosyphilis	16 (21.9)	7 (18.9)	9 (25.0)	
Asymptomatic neurosyphilis	31 (42.5)	16 (43.2)	15 (41.7)	
Penicillin-based therapy, *n* (%)	52 (71.2)	29 (78.4)	23 (63.9)	0.203

Data are shown as median (Q1, Q3) or n (%), unless otherwise indicated. Continuous variables were compared using the Mann–Whitney *U*-test. Categorical variables were compared using the χ^2^ test or Fisher’s exact test, as appropriate. Serologic response was defined as a ≥4-fold decline in serum TRUST titer or seroreversion during follow-up.

CSF, cerebrospinal fluid; MRI, magnetic resonance imaging; OD, optical density; TRUST, toluidine red unheated serum test; WBC, white blood cell.

Patients with a first documented serologic response had higher baseline serum TRUST titers than those without a documented response (median, 1:32 vs. 1:8; *p* < 0.001). CSF TRUST titers were also higher (*p* = 0.020), whereas the difference in CSF WBC count did not reach statistical significance (median, 10.0 vs. 3.0 cells/μL; *p* = 0.052). Baseline salivary OD, CSF protein, clinical subtype, syphilis stage, and treatment regimen did not differ significantly between the groups.

### Characteristics of neuroimaging lesion types and locations

Among the 73 patients, 63 underwent baseline brain MRI. Of these, 36 patients (57.1%) had abnormal neuroimaging findings. The most common lesion type was white-matter lesions or demyelinating-like changes (24/36, 66.7%), followed by cerebral atrophy (16/36, 44.4%) and lacunar infarction, old infarction, or encephalomalacia (10/36, 27.8%). Less frequent findings included inflammatory or encephalitic-like lesions (4/36, 11.1%) and perivascular space or ventricular dilation/hydrocephalus (3/36, 8.3%).

The frontal lobe was the most frequently involved anatomical region (10/36, 27.8%), followed by the thalamus (7/36, 19.4%). Temporal lobe, basal ganglia, brainstem, and periventricular white matter/centrum semiovale/corona radiata involvement were each observed in 4 patients (11.1%). Representative MRI findings are shown in (see Table 2) Figure 1.

**Table 2:** Baseline MRI lesion patterns and anatomical distributions among patients with abnormal neuroimaging findings.

Category/variable	n	Proportion (%)
Lesion type		
White-matter lesions/demyelinating changes	24	66.7
Cerebral atrophy	16	44.4
Lacunar infarction/old infarction/encephalomalacia	10	27.8
Inflammatory or encephalitic-like lesions	4	11.1
Perivascular space or ventricular dilation/hydrocephalus	3	8.3
Lesion location		
Frontal lobe	10	27.8
Thalamus	7	19.4
Temporal lobe	4	11.1
Basal ganglia	4	11.1
Brainstem, midbrain, or pons	4	11.1
Periventricular white matter/centrum semiovale/corona radiata	4	11.1
Parietal lobe	3	8.3
Occipital lobe	2	5.6
Cerebellum	2	5.6
Corpus callosum	2	5.6
Hippocampus	1	2.8

**Figure 1:**
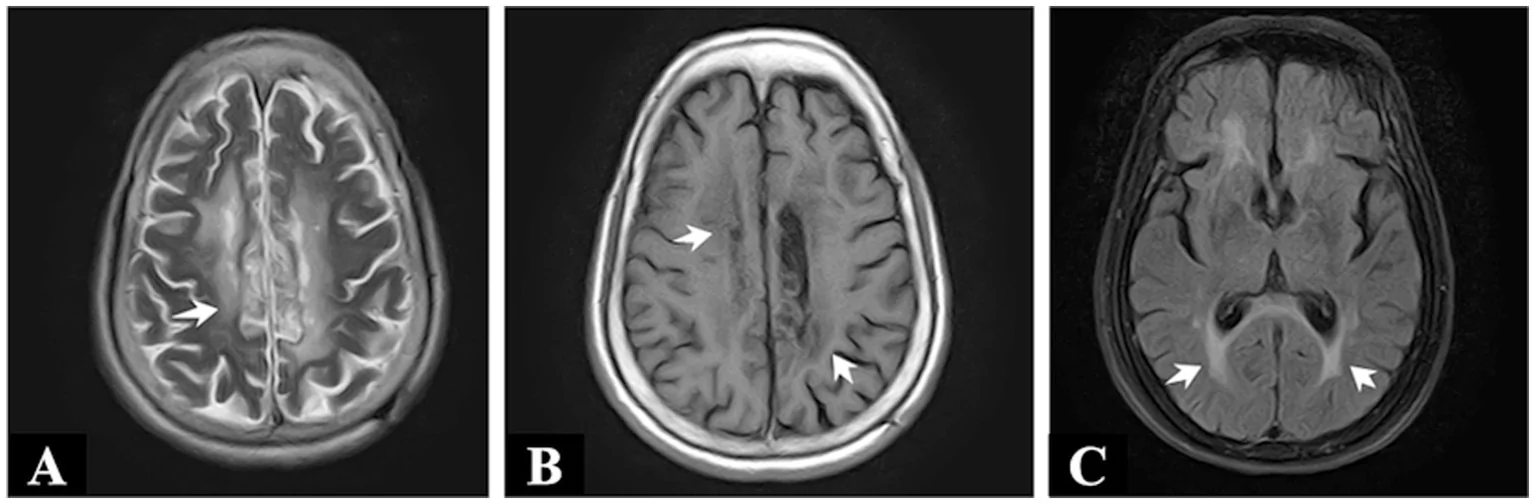
Representative brain MRI findings in a patient with meningovascular neurosyphilis. Axial T2-weighted **(A)**, T1-weighted **(B)**, and FLAIR **(C)** images show multifocal white-matter and ischemic lesions. Arrows indicate representative lesions. FLAIR, fluid-attenuated inversion recovery; MRI, magnetic resonance imaging.

### Kaplan–Meier analyses of the first documented serologic response and CSF normalization

In separate endpoint-specific Kaplan–Meier analyses, the median times to first documented normalization were 12.0 months for CSF TRUST (*n* = 20; 20 events), 13.0 months for CSF WBC (*n* = 15; 12 events), 20.0 months for CSF protein (*n* = 21; 9 events), and 20.0 months for complete CSF normalization (*n* = 27; 12 events) (Figure 2A). Because the curves used different risk sets, the numerical medians do not provide a direct within-patient comparison of biological recovery rates.

**Figure 2:**
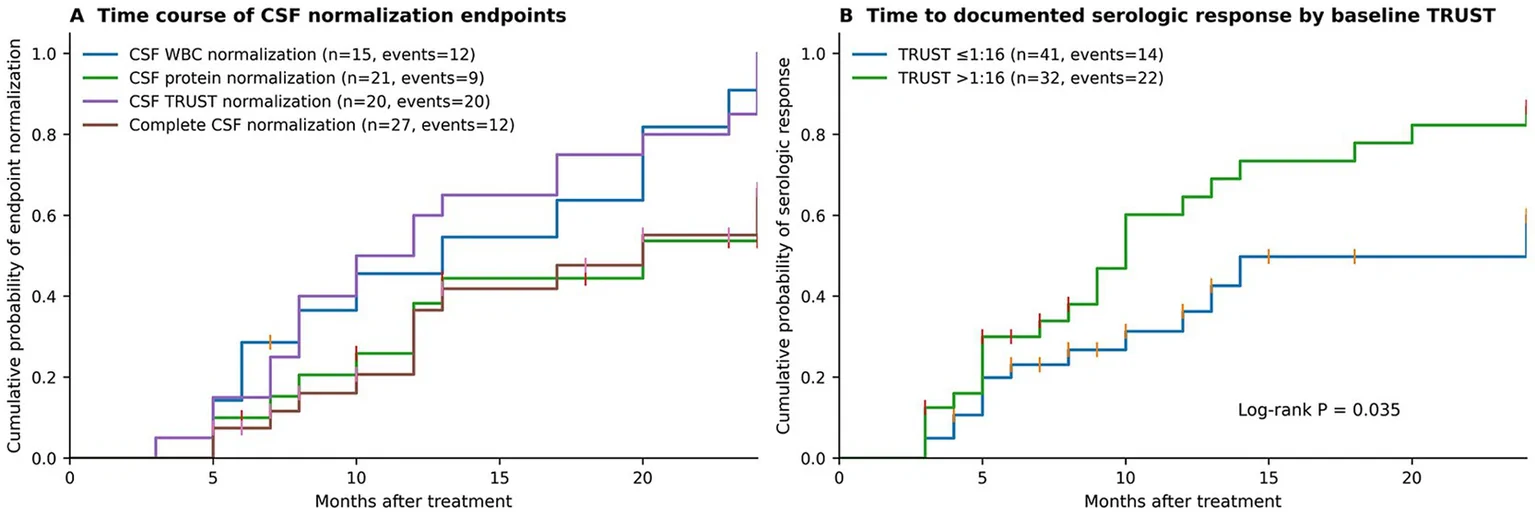
Kaplan–Meier analyses of first documented serologic response and CSF normalization. **(A)** First documented normalization of CSF WBC, protein, TRUST, and all three CSF parameters. The endpoints were analyzed in separate baseline-abnormal risk sets and should not be directly compared as biological recovery rates. **(B)** First documented serologic response according to baseline serum TRUST titer. Tick marks indicate censored observations. CSF, cerebrospinal fluid; TRUST, toluidine red unheated serum test; WBC, white blood cell.

Patients with baseline serum TRUST >1:16 had an earlier first documented serologic response than those with titers ≤1:16 (median, 10.0 vs. 24.0 months; Figure 2B; log-rank *p* = 0.035).

### Salivary antibody dynamics after treatment

Baseline saliva samples were available for 38 patients. Baseline salivary OD did not differ significantly between patients with baseline serum TRUST ≤1:16 and >1:16 (median, 0.35 vs. 0.95; *p* = 0.089; *n* = 38). Among 25 patients with paired baseline and 12-month samples, the salivary OD declined by an estimated 0.36 units (95% bootstrap CI, 0.14–0.64; *p* = 0.011; *q* = 0.017; Figure 3A). The paired cohort comprised 12 patients with baseline TRUST ≤1:16 and 13 patients with TRUST >1:16. In the unadjusted comparison, the higher-titer group had a greater OD decline (Hodges–Lehmann shift, 0.66 units; 95% bootstrap CI, 0.27–1.13; *p* = 0.003; *q* = 0.009; Figure 3B). In the baseline-adjusted sensitivity model, baseline TRUST >1:16 was associated with a lower estimated 12-month OD (adjusted *β*, −0.60 units; 95% CI, −1.17 to −0.02; *p* = 0.044).

**Figure 3:**
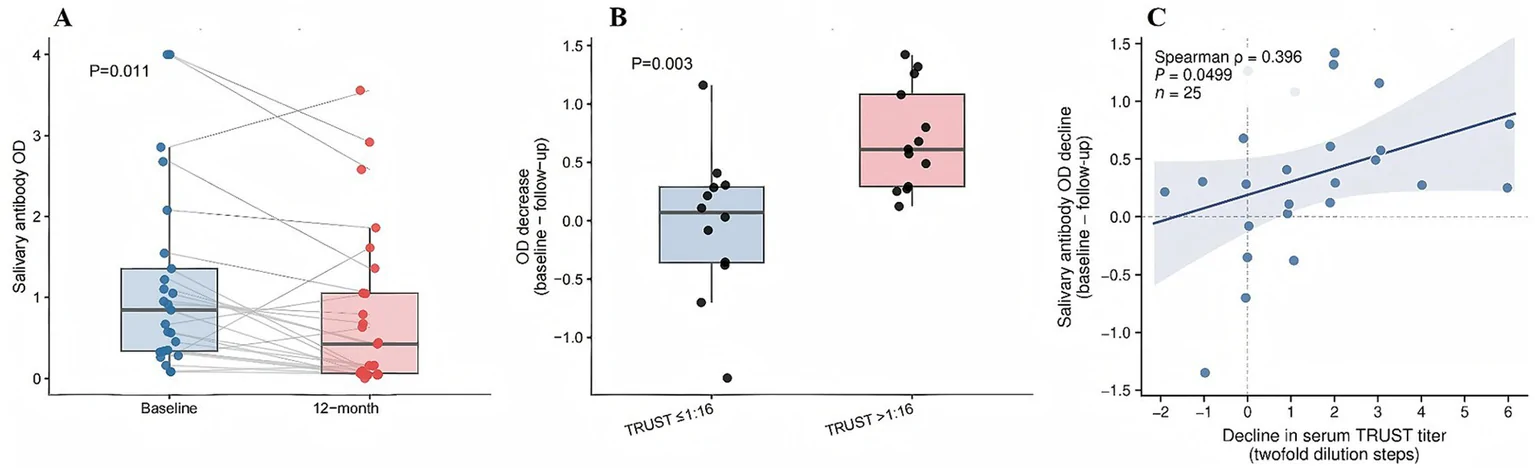
Exploratory longitudinal analyses of salivary anti-*Treponema pallidum* antibody OD. **(A)** Paired baseline and 12-month OD signals (*n* = 25; Hodges–Lehmann decrease, 0.36 OD units; 95% bootstrap CI, 0.14–0.64; *p* = 0.011; *q* = 0.017). **(B)** OD decline according to baseline serum TRUST titer (≤1:16, *n* = 12; >1:16, *n* = 13). The unadjusted between-group shift was 0.66 OD units (95% bootstrap CI, 0.27–1.13; *p* = 0.003; *q* = 0.009). After adjustment for baseline OD, the higher-TRUST group had a lower estimated 12-month OD (adjusted difference, −0.60 OD units; 95% CI, −1.17 to −0.02; *p* = 0.044). **(C)** Correlation between salivary OD decline and serum TRUST decline (*n* = 25; Spearman *ρ* = 0.396; 95% bootstrap CI, 0.034–0.665; *p* = 0.0499; *q* = 0.050). The fitted line and confidence band are shown for visualization only. The q values were calculated using the Benjamini–Hochberg procedure across the three primary analyses; the baseline-adjusted model was a sensitivity analysis of panel **B**. OD, optical density; TRUST, toluidine red unheated serum test.

Salivary OD decline correlated modestly with serum TRUST decline (Spearman *ρ* = 0.396; 95% bootstrap CI, 0.034–0.665; *p* = 0.0499; *q* = 0.050; *n* = 25; Figure 3C). Baseline characteristics of patients with paired saliva samples and the remainder of the cohort are compared in Supplementary Table S4.

### Cox regression analyses for the time to serologic response

The final exploratory multivariable Cox model included all 73 patients and 36 events (Figure 4). Higher baseline serum TRUST titer was associated with an earlier first documented serologic response in univariable analysis (HR per twofold increase, 1.33; 95% CI, 1.12–1.58; *p* = 0.001) and after adjustment (adjusted HR, 1.34; 95% CI, 1.11–1.61; *p* = 0.002). Elevated CSF protein was directionally associated with a later documented response in univariable analysis (HR, 0.51; 95% CI, 0.25–1.02) and in the multivariable model (adjusted HR, 0.41; 95% CI, 0.20–0.86; *p* = 0.017). Symptomatic status and penicillin-based therapy were not significantly associated with the endpoint. The global Schoenfeld residual test did not indicate an overall proportional-hazard violation (χ^2^ = 7.542; df = 4; *p* = 0.110).

**Figure 4:**
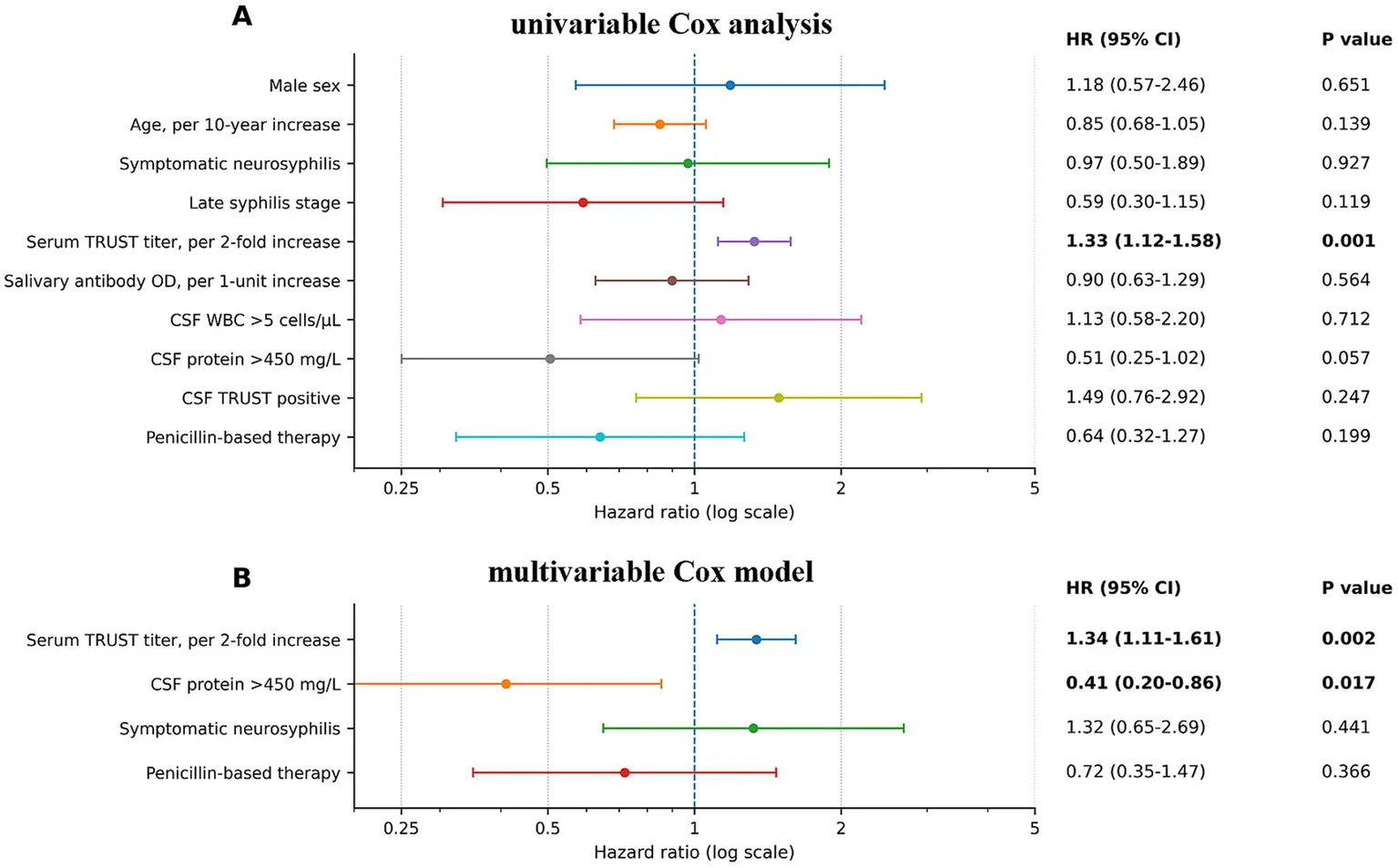
Cox regression analyses of time to first documented serologic response. **(A)** Univariable analyses of candidate baseline variables. **(B)** Exploratory multivariable model including baseline serum TRUST titer, elevated CSF protein, symptomatic neurosyphilis, and penicillin-based therapy (*n* = 73; 36 events). Baseline serum TRUST was modeled per twofold increase; HR > 1 indicates earlier documentation of response. CI, confidence interval; CSF, cerebrospinal fluid; HR, hazard ratio; OD, optical density; TRUST, toluidine red unheated serum test; WBC, white blood cell.

### Descriptive follow-up MRI findings

Among the 13 clinically selected patients with follow-up MRI, imaging findings remained stable in nine (69.2%). Chronic structural abnormalities, including cerebral atrophy, lacunar infarction, encephalomalacia, and longstanding white-matter lesions, generally persist. Partial or complete resolution of white-matter or inflammatory-like lesions was observed in two patients, where one developed a new focal lesion and another developed a new lacunar infarct. Given the small, clinically selected sample and non-standardized imaging schedule, these findings are descriptive, and no inferential association with serologic or CSF response was assessed. Individual neuroimaging changes are summarized in Table 3.

**Table 3:** Descriptive MRI changes in 13 patients with follow-up imaging.

No.	Clinical subtype/stage	Serologic response	CSF normalization	Baseline MRI pattern	Follow-up MRI change
1	Asymptomatic neurosyphilis/late	No	Partial normalization (CSF-TRUST)	Normal	Normal
2	Tabes dorsalis/late	Yes	Partial normalization (CSF-TRUST)	White-matter lesions with mild cerebral atrophy	Unchanged
3	Asymptomatic neurosyphilis/late	No	No	Mild white-matter demyelinating changes with mild cerebral atrophy	Unchanged
4	Tabes dorsalis/late	Yes	Partial normalization (CSF-TRUST)	Cerebral atrophy with white-matter demyelination	Unchanged
5	General paresis/late	No	No	White-matter lesions with mild cerebral atrophy	Unchanged
6	Meningovascular neurosyphilis/late	Yes	Partial normalization (CSF-TRUST)	Normal	Normal
7	Asymptomatic neurosyphilis/late	No	No	Normal	New small abnormal signal adjacent to the anterior horn of the right lateral ventricle
8	Asymptomatic neurosyphilis/late	Yes	Partial normalization (CSF-WBC)	Normal	Normal
9	Meningovascular neurosyphilis/early	No	Partial normalization (CSF-WBC)	Multifocal supra- and infratentorial infarcts with diffuse periventricular white-matter degeneration and cerebral atrophy	No marked improvement
10	Asymptomatic neurosyphilis/late	Yes	Complete normalization	Mild bilateral frontal white-matter lesions	Frontal-lobe abnormal signals decreased
11	Meningitic neurosyphilis/early	Yes	Complete normalization	Patchy hyperintense lesions in the left frontal and occipital lobes and the right parietal lobe	Abnormal signals disappeared
12	Meningitic neurosyphilis/early	Yes	Complete normalization	Normal	Normal
13	General paresis/late	Yes	Complete normalization	White-matter demyelinating lesions with cerebral atrophy	New lacunar infarct in the splenium; chronic white-matter lesions unchanged

CSF, cerebrospinal fluid; FLAIR, fluid-attenuated inversion recovery; MRI, magnetic resonance imaging; TRUST, toluidine red unheated serum test; WBC, white blood cell.

## Discussion

This exploratory retrospective cohort characterized post-treatment serologic, CSF, salivary antibody, and MRI findings in HIV-negative neurosyphilis (15, 17). The novel finding was a decline in salivary anti-*Treponema pallidum* antibody OD signals at 12 months and their modest correlation with serum TRUST decline, providing preliminary evidence of a treatment-associated salivary signal.

This study provides longitudinal evidence that salivary anti-*T. pallidum* antibody OD signals decrease after treatment. The baseline-adjusted analysis and the modest correlation with serum TRUST decline support the biological relevance of this signal. Together, these findings support further evaluation of salivary antibody OD as a non-invasive measure of treatment-associated change. However, the limited paired sample and lack of quantitative assay validation preclude conclusions regarding individual-level discrimination or added value over serum TRUST. Prospective studies with standardized sampling, validated quantitative assays, and predefined serologic, CSF, and clinical endpoints are warranted.

Approximately half of the cohort achieved a first documented serologic response. In the separate CSF risk sets, CSF TRUST and WBC showed earlier numerical medians for first documented normalization than CSF protein and complete CSF normalization. However, irregular lumbar puncture schedules resulted in interval-censored event times, and the different risk sets precluded direct comparison of biological recovery rates. These observations are consistent with, but do not independently establish, previously reported differences in CSF follow-up patterns after neurosyphilis treatment (13, 15).

CSF protein is non-specific and may reflect blood–brain barrier dysfunction, residual intrathecal inflammation, or chronic structural injury rather than persistent active neurosyphilis (18, 19). Although elevated baseline CSF protein was associated with a longer time to first documented serologic response, this exploratory finding should be interpreted cautiously given the limited number of events and requires confirmation in larger studies.

Higher baseline serum TRUST titers were associated with earlier first documented serologic response. Because response was defined as a fourfold decline or seroreversion, baseline titer was mathematically linked to the endpoint and influenced the opportunity to meet it. This association is therefore definition-dependent and should not be interpreted as evidence of a better biological treatment response at higher baseline titers.

Baseline MRI demonstrated heterogeneous abnormalities consistent with previous reports of white-matter lesions, cerebral atrophy, infarction, and inflammatory changes in neurosyphilis (20, 21). Among 13 clinically selected patients with repeat MRI, some white-matter or inflammatory-like lesions improved, whereas chronic structural abnormalities generally remained stable. Because repeat imaging was not systematically scheduled and the sample was small, these findings are descriptive and exploratory and cannot establish a relationship between radiological changes and treatment response. MRI findings should therefore be interpreted alongside clinical, serological, and CSF assessments.

This study has several limitations. It was a single-center retrospective study with a modest sample size. Follow-up serum testing, lumbar punctures, saliva collection, and MRI examinations were clinically determined rather than standardized, introducing interval censoring, informative observation, timing bias, and selection bias. Complete visit-level bracketing information was not uniformly available, precluding reliable interval-censored sensitivity analyses. CSF curves arose from different endpoint-specific risk sets and cannot be compared with biological normalization rates. Paired saliva was available for only 25 patients, and the commercial ELISA was not quantitatively validated for longitudinal saliva measurements. The Cox model contained only 36 events for four covariates, making the CSF-protein estimate potentially unstable.

In conclusion, salivary anti-*T. pallidum* antibody OD decreased 12 months after treatment, supporting a preliminary treatment-associated salivary signal. Higher baseline serum TRUST was associated with an earlier first documented serologic response, although this relationship was partly definition-dependent. CSF and MRI findings require cautious interpretation. Prospective studies with standardized follow-up and quantitatively validated salivary assays are warranted.

## Data Availability

The original contributions presented in the study are included in the article/Supplementary material, further inquiries can be directed to the corresponding author.
