# Supplementary material for: Post-treatment serologic, cerebrospinal fluid, MRI, and salivary antibody responses in HIV-negative neurosyphilis: a retrospective study
Source: Front Neurol. 2026 Sep 29;17:1939496. doi: 10.3389/fneur.2026.1939496 (PMC13657999; doi:10.3389/fneur.2026.1939496)
Supplement: Supplementary file 1 [file Table_1.DOCX]

**Supplementary Material**

*Post-treatment Serologic, Cerebrospinal Fluid, MRI, and Salivary Antibody Responses in HIV-negative Neurosyphilis: a retrospective study*

**Supplementary Table S1. Number of evaluable patients and timing of endpoint-defining post-treatment assessments for each endpoint**

| **Endpoint** | **Patients, n** | **Events, n** | **Censored, n** | **Endpoint-defining or censoring assessment time, median (IQR), months** | **Range, months** | **≤6 months** | **>6–12 months** | **>12–18 months** | **>18–24 months** |
| --- | --- | --- | --- | --- | --- | --- | --- | --- | --- |
| First documented serum TRUST response | 73 | 36 | 37 | 8.0 (4.0–12.0) | 3–24 | 33 | 22 | 8 | 10 |
| First documented CSF WBC normalization | 15 | 12 | 3 | 8.0 (6.0–18.5) | 3–24 | 5 | 4 | 2 | 4 |
| First documented CSF protein normalization | 21 | 9 | 12 | 12.0 (8.0–20.0) | 3–24 | 4 | 7 | 4 | 6 |
| First documented CSF TRUST normalization | 20 | 20 | 0 | 11.0 (7.8–17.8) | 3–24 | 3 | 9 | 3 | 5 |
| First documented complete CSF normalization | 27 | 12 | 15 | 12.0 (7.5–19.0) | 5–24 | 5 | 10 | 5 | 7 |

**Note:** For patients with an event, assessment time refers to the first assessment documenting the endpoint; for censored patients, it refers to the last available assessment. Counts in the four follow-up windows represent patients, with each patient contributing once per endpoint. Complete counts of all repeated assessments were not uniformly available. CSF, cerebrospinal fluid; IQR, interquartile range; TRUST, toluidine red unheated serum test; WBC, white blood cell.

**Supplementary Table S2. Clinical and cerebrospinal fluid findings supporting the diagnosis of neurosyphilis**

| **Diagnostic finding or profile** | **Symptomatic patients (n=42), n (%)** | **Asymptomatic patients (n=31), n (%)** | **Total (n=73), n (%)** |
| --- | --- | --- | --- |
| Reactive CSF TPPA | 41 (97.6) | 30 (96.8) | 71 (97.3) |
| Nonreactive CSF TPPA | 1 (2.4) | 1 (3.2) | 2 (2.7) |
| Reactive CSF TRUST | 22 (52.4) | 10 (32.3) | 32 (43.8) |
| Nonreactive CSF TRUST | 20 (47.6) | 21 (67.7) | 41 (56.2) |
| CSF WBC >5 cells/μL | 19 (45.2) | 15 (48.4) | 34 (46.6) |
| CSF protein >450 mg/L | 30 (71.4) | 18 (58.1) | 48 (65.8) |
| At least one CSF inflammatory abnormality | 33 (78.6) | 25 (80.6) | 58 (79.5) |
| **Mutually exclusive diagnostic profiles** |  |  |  |
| Reactive CSF TRUST | 22 (52.4) | 10 (32.3) | 32 (43.8) |
| Nonreactive CSF TRUST with CSF inflammation | 11 (26.2) | 18 (58.1) | 29 (39.7) |
| Nonreactive CSF TRUST without defined CSF inflammation | 9 (21.4) | 0 | 12 (16.4) |
| Nonreactive CSF TRUST without defined CSF inflammation or compatible manifestations, with CSF TPPA titer ≥1:320 | 0 | 3 (9.7) | 3 (4.1) |

**Note:** Percentages were calculated using the number of patients in each column as the denominator. CSF inflammation was defined as a CSF WBC count >5 cells/μL and/or a CSF protein concentration >450 mg/L. The profiles were defined hierarchically according to CSF TRUST reactivity and, among patients with nonreactive CSF TRUST, the presence or absence of CSF inflammation. Individual findings were interpreted as part of the overall clinical assessment and were not considered independently diagnostic. CSF, cerebrospinal fluid; TPPA, Treponema pallidum particle agglutination assay; TRUST, toluidine red unheated serum test; WBC, white blood cell.

**Supplementary Table S3. Availability and missingness of candidate covariates for Cox regression**

| **Candidate covariate** | **Coding or scale used in analysis** | **Available, n (%)** | **Missing, n (%)** | **Univariable Cox analysis** | **Final multivariable Cox model** |
| --- | --- | --- | --- | --- | --- |
| Sex | Male vs. female | 73 (100.0) | 0 (0.0) | Included | Not included |
| Age | Per 10-year increase | 73 (100.0) | 0 (0.0) | Included | Not included |
| Symptomatic neurosyphilis | Symptomatic vs. asymptomatic | 73 (100.0) | 0 (0.0) | Included | Included |
| Syphilis stage | Late vs. early syphilis | 73 (100.0) | 0 (0.0) | Included | Not included |
| Baseline serum TRUST titer | log₂-transformed; per twofold increase | 73 (100.0) | 0 (0.0) | Included | Included |
| Baseline salivary antibody OD | Per 1-unit increase | 38 (52.1) | 35 (47.9) | Included | Not included |
| Baseline CSF WBC count | >5 vs. ≤5 cells/μL | 73 (100.0) | 0 (0.0) | Included | Not included |
| Baseline CSF protein concentration | >450 vs. ≤450 mg/L | 73 (100.0) | 0 (0.0) | Included | Included |
| Baseline CSF TRUST | Reactive vs. nonreactive | 73 (100.0) | 0 (0.0) | Included | Not included |
| Treatment regimen | Penicillin-based vs. non-penicillin-based therapy | 73 (100.0) | 0 (0.0) | Included | Included |
| Baseline brain MRI abnormality | Abnormal vs. normal | 63 (86.3) | 10 (13.7) | Not modeled | Not included |

**Note:** The final complete-case multivariable model included all 73 patients and 36 first documented serologic-response events. All four modeled covariates were complete, and no imputation was performed. Baseline salivary antibody OD was analyzed using available cases in the univariable analysis. MRI variables were not modeled. CSF, cerebrospinal fluid; MRI, magnetic resonance imaging; OD, optical density; TRUST, toluidine red unheated serum test; WBC, white blood cell.

**Supplementary Table S4. Baseline comparison of patients with paired saliva samples and the remainder of the cohort**

| **Baseline characteristic** | **Patients with paired saliva samples (n=25)** | **Remaining cohort (n=48)** | **P value** |
| --- | --- | --- | --- |
| Age, years | 42.0 (28.0–57.0) | 37.0 (30.5–53.3) | 0.675 |
| Male sex, n (%) | 15 (60.0) | 36 (75.0) | 0.282 |
| Symptomatic neurosyphilis, n (%) | 14 (56.0) | 28 (58.3) | 1.000 |
| Late syphilis stage, n (%) | 11 (44.0) | 28 (58.3) | 0.324 |
| Baseline serum TRUST titer | 1:32 (1:8–1:32) | 1:16 (1:8–1:32) | 0.506 |
| Baseline salivary antibody OD¹ | 0.85 (0.34–1.36) | 0.88 (0.05–1.74) | 0.601 |
| Reactive CSF TRUST, n (%) | 11 (44.0) | 21 (43.8) | 1.000 |
| CSF WBC count >5 cells/μL, n (%) | 11 (44.0) | 23 (47.9) | 0.808 |
| CSF protein concentration >450 mg/L, n (%) | 15 (60.0) | 33 (68.8) | 0.604 |
| Penicillin-based therapy, n (%) | 19 (76.0) | 33 (68.8) | 0.594 |
| Abnormal baseline brain MRI², n/N (%) | 11/23 (47.8) | 25/40 (62.5) | 0.298 |

**Note:** Data are presented as median (interquartile range) or n (%), unless otherwise indicated. Continuous variables were compared using the Mann–Whitney U test, and categorical variables were compared using Fisher’s exact test. Serum TRUST titers were converted to twofold dilution steps for statistical comparison. All P values are two-sided and unadjusted. These comparisons were exploratory; the absence of statistically significant differences should not be interpreted as evidence of equivalence between the groups. ¹Baseline salivary OD was available for 25 patients in the paired-saliva group and 13 patients in the remaining cohort. ²Baseline MRI was available for 23 and 40 patients, respectively. CSF, cerebrospinal fluid; MRI, magnetic resonance imaging; OD, optical density; TRUST, toluidine red unheated serum test; WBC, white blood cell.
